# A multi-faceted discovery strategy identifies functional antibodies binding to cysteine-rich domain 1 of hDKK1 for cancer immunotherapy via Wnt non-canonical pathway

**DOI:** 10.1038/s41388-025-03445-6

**Published:** 2025-05-20

**Authors:** Linya Wang, Sean M. Peterson, Marisa Yang, Ana G. Lujan Hernandez, Tom Z. Yuan, Zhen Han, Vishwas Prabhu, Kara Y. Chan, Cameron F. Hu, Mouna Villalta, Tammy Htoy, Paul VanDyke, Carson Holliday, Hector Franco, Hansika Wadhwa, Hoa Giang, Ryan Stafford, Fumiko Axelrod, Aaron Sato

**Affiliations:** Twist Bioscience, South San Francisco, CA USA

**Keywords:** Drug discovery, Cancer

## Abstract

Wnt signaling is important in embryonic development and tumorigenesis. These biological effects can be exerted by the activation of the β-catenin-dependent canonical pathway or the β-catenin-independent non-canonical pathway. DKK1 is a potent inhibitor of Wnt signaling by competing with Wnt binding to LRP5/6 co-receptors. DKK1 is tumorigenic in multiple cancer types and immunosuppressive in NK cells. Emerging evidence indicates that DKK1 is involved in T cell differentiation and induces cancer evasion of immune surveillance by accumulating MDSCs. Consequently, DKK1 has become a promising target for cancer immunotherapy, and the mechanisms by which DKK1 affects cancer and immune cells have received considerable attention. Using Twist’s precision DNA writing technologies, we created phage display libraries with a diversity greater than 1 × 10^10^ individual members, and machine learning models were utilized for optimal discovery. We found that anti-DKK1 antibodies blocked the binding of DKK1 to LRP co-receptors. Binding of antibodies to different cysteine-rich domains (CRDs) of hDKK1 leads to different activation effects. In vitro functional assays showed that the interaction of Wnt with LRP5/6 co-receptors was restored in the presence of anti-DKK1 antibodies binding to DKK1 C-terminal CRD2, resulting in the upregulation of Wnt canonical TCF/LEF signaling and reactivation of osteoblast differentiation. Moreover, anti-DKK1 antibodies binding to DKK1 N-terminal CRD1 induced Wnt non-canonical JNK phosphorylation, immune cell activation, and tumor cell cytotoxicity. In vivo studies indicated that these anti-DKK1 antibody leads targeting DKK1 CDR1 are potent in inhibition of tumor growth and may have promising efficacy as cancer immunotherapy due to activation of the Wnt non-canonical pathway.

## Introduction

The immune system regulates the development, growth, and metastasis of tumors. While the immune system actively works to inhibit these processes, tumors evolve sophisticated evasion mechanisms to circumvent immune-mediated elimination by reducing their ability to be recognized as different from non-transformed cells (antigenicity) and their ability to provoke an immune response (immunogenicity) [1]. Various immunotherapeutic approaches have proven successful in the clinic, ranging from immune checkpoint inhibitors targeting programmed death-ligand 1 (PD-L1) and cytotoxic T-lymphocyte associated protein 4 (CTLA-4) to chimeric antigen receptor T (CAR-T) cells. However, only a minority of cancer patients experience durable responses to these therapeutics [2]. Alternative approaches are needed to increase response rates and buttress current immunotherapeutic strategies to enhance their clinical efficacy.

The Wingless-related integration site (Wnt) family constitutes a large group of secreted lipid-modified signaling glycoproteins. They are involved in various developmental processes such as embryonic patterning, cell growth, migration, and differentiation [3]. Mutations in Wnt pathway components are associated with human diseases, including cancer [4]. The Wnt signaling pathway involves several receptors and co-receptors, including Frizzled (FZD) receptors, low-density lipoprotein receptor-related protein (LRP), receptor tyrosine kinase-like orphan receptor (ROR), and its related receptor tyrosine kinase (RYK) receptor. Wnt pathways are divided into two main branches: ‘canonical’ or β-catenin-dependent, and ‘non-canonical’ or β-catenin-independent pathways. The β-catenin-dependent pathway involves several Wnt ligands (e.g., WNT1, WNT3A, and WNT8), FZDs, and LRP5/6 receptors, leading to the inhibition of glycogen synthase kinase 3β (GSK-3β). This results in the loss of β-catenin phosphorylation, preventing its degradation by the proteasome, causing its accumulation in the cytoplasm and translocation into the nucleus, where it binds to T-cell factor (TCF)/lymphoid enhancer-binding factor (LEF) and activates the transcription of Wnt target genes [5]. In contrast, the Wnt non-canonical pathway is subdivided into planar cell polarity (PCP) and Ca^2+^-dependent pathways. The Wnt/PCP pathway signals via RhoA, Rac GTPases, and c-Jun N-terminal kinase (JNK). The Wnt/Ca^2+^ pathway involves G-proteins, the nuclear factor of activated T-cells, Ca^2+^/calmodulin-dependent protein kinase II, and protein kinase C. It is well established that the non-canonical pathway antagonizes the functions of canonical ligands [6].

The secreted Wnt inhibitor Dickkopf-1 (DKK1) has emerged as a promising target for immunotherapeutic intervention [7]. Dickkopf-1 antagonizes the Wnt co-receptor low-density lipoprotein receptor–related protein 5/6 (LRP5/6) [8] and, therefore, the Wnt–β-catenin T cell-specific factor (TCF) canonical signaling pathway. DKK1 has been identified as both a tumor suppressor and metastasis promoter. Despite this controversy, recent evidence points to an immunomodulatory role for DKK1 in the tumor microenvironment [7]. In Lewis lung carcinoma and melanoma mouse models, antibody-mediated neutralization of DKK1 reduced tumor growth and myeloid-derived suppressor cell (MDSC) accumulation in a β-catenin-dependent manner [9]. Treatment with the humanized immunoglobulin G4 (IgG4) anti-DKK1 antibody, DKN-01, inhibited tumor growth in a natural killer (NK) cell-dependent manner in both a metastatic breast cancer model [10] and a prostate cancer model [11]. Together, these data demonstrate that DKK1 is a driver of immune evasion in several cancers and represents an attractive target for immunotherapeutic discovery. The discovery of antibodies targeting DKK1 is a potential strategy for cancer immunotherapy.

Phage display has been used to discover several immunotherapeutic antibodies such as avelumab (Bavencio^®^), atezolizumab (Tecentriq™), and relatlimab (Opdualag™) [12]. This method involves inserting exogenous DNA fragments (antibody genes) into filamentous phage coat protein genes so that they can be expressed in filamentous phages and their gene products (antibody fragments) are subsequently enriched for antigen binding by phage panning, enabling libraries of tens of billions of antibodies to be screened. Advances in gene synthesis have increased the scale and precision of antibody library construction [13–15]; however, designing *high-quality* libraries that are rich in functional, developable antibodies for synthesis at this scale remains challenging. Moreover, the impracticality of triaging thousands of candidates after biopanning often risks culling many, if not most, high-affinity candidates at the beginning of the screening process. Machine learning, with its ability to recognize non-obvious patterns in massive datasets, has the potential to alleviate these bottlenecks by informing library design and facilitating candidate prioritization [16]. Machine learning approaches have already been leveraged to design finessed antibody libraries with optimized developability, epitope specificity, and target affinity [17–20].

In this report, we describe the discovery and characterization of antagonistic antibodies targeting hDKK1. To maximize the success of this discovery campaign, we prepared several synthetic VHH phage libraries with advanced machine learning clone selection for greater variation. This comprehensive discovery campaign ultimately yielded specific, high-affinity, cross-reactive, and humanized antibody antagonists of DKK1. Antibodies binding to DKK1 C-terminal cysteine-rich domain 2 (CRD2) restored the Wnt canonical pathway in TCF/LEF signaling and osteoblast differentiation, and binding to the DKK1 N-terminal CRD1 restored the Wnt non-canonical pathway by activating JNK phosphorylation, promoting a robust immune response, and demonstrating in vivo tumor suppressing activity.

## Materials and methods

### Construction of synthetic anti-DKK1 antibody libraries

Four libraries were generated for screening against human DKK1: one VHH synthetic library and three VHH machine learning synthetic libraries (Table 1). CDR diversities for all libraries were screened to remove sequences that may cause manufacturing issues, such as those linked to post-translational modifications, cryptic splice sites, and commonly used nucleotide restriction enzyme sites. The resulting sequences were then encoded using oligonucleotide pools synthesized by Twist Bioscience. For the VHH library, a partially humanized framework was used by retaining the llama FW2 region within the human DP-47 framework to maintain stable expression as a heavy chain-only antibody. The VHH cassette was subsequently cloned into the pADL-22c phagemid display vector (Antibody Design Labs) by SfiI restriction digestion and electroporated into TG1 *E. coli* cells (Lucigen). The purified phage library was estimated to have a diversity of 1.0 × 10^10^, as determined by a dilution series of colony-forming units per mL on 2YT agar plates containing 100 μg/mL carbenicillin.

**Table 1:** Summary of libraries and triage results for lead identification.

Project	Library	No. clones reformatted for primary assays	TCF/LEF functional assay hits	Primary immune cell activators	Tumor killer
SC-52	VHH hShuffle/Hyperimmune	113	5	4	4
TB643	ML Approach	97	12	8	6
	(VHH hShuffle/HI)				
	KNN based classifier				
TB758	ML Approach	170	7	7	5
	(VHH hShuffle/HI)				
	CNN based neural network				

The VHH hShuffle and hShuffle HI library series have a partially humanized framework which lowers the immunogenicity of the sequences isolated from the library for drug development. Specifically, framework 1, 3, and 4 are humanized. Framework 2 is a camelid framework to allow for better stability of the library. With the help of our in-house oligo printing at Twist, we have each CDR sequence individually synthesized instead of using degenerate codons. Both of these libraries are combinatorically generated which gives us a diversity of ~e9–e10. The hShuffle library has natural llama CDR sequences for CDR 1, 2, and 3. The hShuffle HI library has llama CDR sequences for CDR 1 and 2 and human CDR sequences for CDR3. This increases the diversity of CDR3 ~ 2.5 million sequences. These 2.5 million sequences are derived from human naive and memory B-cell sequences. This significantly increases the diversity of the library. The unique sequences from phage ELISA screening were selected for reformatting, production, and assay characterization.

### Phage panning and screening strategy to isolate DKK1 binding clones

The phage particles were blocked with phosphate-buffered saline (PBS) containing 5% bovine serum albumin (BSA), and nonspecific binders were removed using M-280 streptavidin-coated magnetic beads (ThermoFisher Scientific). Biotinylated DKK1 protein (Acro, #DK1–H82F5) was mixed with M-280 beads (100 nM per 1 mg of beads) and washed with PBS/0.5% Tween 20 to remove unbound proteins. The mixture was then used as an antigen target for four rounds of phage selection. The phage supernatant depleted of nonspecific binders was transferred to bead mixtures containing bound biotinylated DKK1 and allowed to bind for 1 h at room temperature with gentle rotation to select for binders. After incubation, the beads were washed several times with PBS/0.5% Tween 20 to remove non-binding and low-affinity clones. The remaining bound phages were eluted with trypsin (10 mg/mL) in PBS for 30 min at 37 °C. The output supernatant enriched with binding clones was amplified from TG1 *E. coli* cells for the next round of selection. For each subsequent round, the number of wash cycles was increased incrementally, and the concentration of antigen was reduced to select higher affinity DKK1 binders.

Bacterial colonies containing the phagemid display vector were isolated on 2YT agar plates containing 100 µg/mL carbenicillin. Single colonies were selected using QPix 420 (Molecular Devices, Sunnyvale, CA, USA) in 384-well plates containing 2YT and M13KO7 helper phage (Antibody Design Labs). Phage ELISAs were conducted using Nunc 384-well plates (ThermoFisher Scientific) with passively absorbed DKK1 or BSA proteins at 2 µg/mL. Anti-M13 antibody conjugated to horseradish peroxidase (HRP) (Sino Biological) was used to detect the presence of bound phages following the addition of a 3,3ʹ,5,5ʹ-tetramethylbenzidine (TMB) substrate. Clones that demonstrated three-fold binding over a BSA background were subjected to rolling circle amplification (RCA) and Sanger sequencing of GENEWIZ. The VHH regions were identified using PhiS4 (GCGGATAACAATTTGAATTCAAGGAGACAG).

### Next-generation sequencing (NGS) analysis

Phagemid DNA was miniprepped from the output bacterial stocks of all panning rounds. VHH DNA sequences were PCR amplified from the phagemid DNA. The PCR product was directly used for library preparation using a KAPA HyperPlus Library Preparation Kit (Kapa Biosystems, #KK8514). To add diversity to the library, the samples were spiked with 15% PhiX Control (Illumina, #FC-110-3001). The library was then loaded onto Illumina’s 600 cycle MiSeq Reagent Kit v3 (Illumina, #MS-102-3003) and analyzed using the MiSeq instrument.

### Clone selection

A K-nearest neighbors (KNN) classification algorithm was employed to select TB643 clones (ML strategy) from the corresponding panning output. Briefly, positive and negative classes were defined using clones from the previous SPR binding data to train the model. Sequence-based descriptors were computed and normalized, followed by PCA dimensionality reduction. After hyperparameter tuning for PCA components and the number of neighbors optimizing the F-beta score, clones were identified from NGS sequencing from round 4 based on predicted binding obtained from the KNN classifier.

**Fig. 1:**
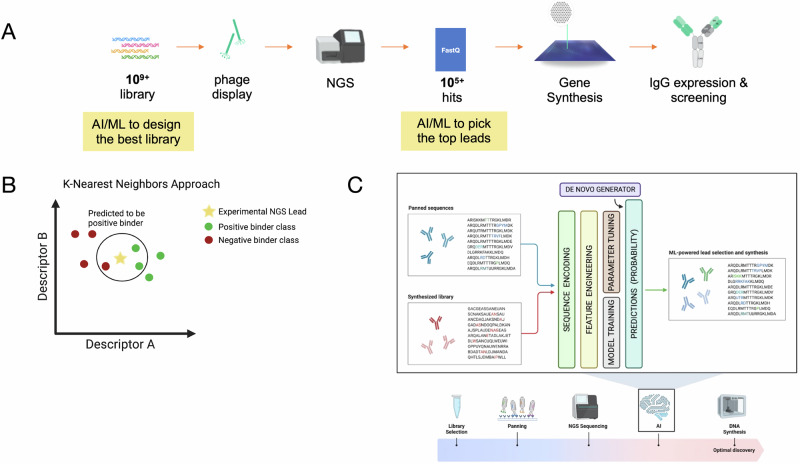
Machine learning strategy. **A** The objectives of Twist machine learning platform are to obtain a higher yield of hit clones with highest affinity, greatest sequence diversity, and the best biophysical properties. **B** KNN based classifier trained based on previous SPR binding data. **C** CNN based neural network used to train on previously identified leads and NGS data to predict binders.

Two main methods were used to select the final TB758 clones for reformatting: a Convolutional Neural Network (CNN) and an enrichment workflow. For the CNN approach, positive and negative classes were defined using clones from previous SPR binding data, supplemented with NGS variants. Enriched clones from the original panning output (round 4) were used to supplement the positive class, while a random sample of the initial library was used for the negative class. After training and selecting the best performing model (based on the F1 score selected via the validation set), round four NGS variants were scored, and the predicted probability from the CNN was used to threshold potential binders from non-binders. The predicted binders were then run using an unsupervised learning algorithm (clustering) to generate a diverse set of candidates. For the enrichment workflow, Immunarch software [21] was used to identify the top enriched clonotypes from rounds 3 to 4, followed by a custom pipeline for selecting the top three most abundant variants for each clonotype. The variants identified by the two methods were aggregated and unique variants were selected for reformatting (Fig. 1).

### Reformatting, expression, and purification of monoclonal antibodies

VHHs were reformatted to human IgG2 for DNA back-translation, synthesis, and cloning into the mammalian expression vector pTwist CMV BG WPRE Neo using the Twist Bioscience eCommerce portal. Clonal genes were delivered as purified plasmid DNA ready for transient transfection into HEK Expi293 cells (ThermoFisher Scientific). Antibodies selected for in vitro cell-based assays were expressed in 8 mL or 30 mL cultures using the same expression system for larger production. Cultures were grown for 4–5 days, harvested, and purified with Phynexus Protein A resin tips with Cytiva PrsimA resin on Hamilton Microlab STAR automated liquid-handling systems. Purified antibodies were concentrated using Amicon and 30 kDa cut-off spin filters. For in vivo evaluation, antibodies were expressed in 300 mL cultures, grown for 5 days, harvested, and purified using Bio-work TREN 40 pre-packed columns for low endotoxin purification, followed by a Cytiva PrismA pre-packed column on the AKTA Pure system. The antibodies were eluted with 50 mM citrate buffer (pH 3.0) and neutralized to a final concentration of 148 mM HEPES (pH 6.5). The neutralized eluted IgG2 samples were dialyzed against 1x Dulbecco’s phosphate-buffered saline (DPBS, pH 7.4), overnight, and changed to fresh cold 1x DPBS (pH 7.4) once in-between. CE-SDS was used to determine antibody purity and confirm the molecular weight for all scales of expression cultures. The IgGs used for in vivo studies were further characterized by size exclusion chromatography with high-performance liquid chromatography (Thermo Ultimate 3000). Endotoxin levels were tested for all materials used in the animal study (Endosafe® nexgen-PTSTM Endotoxin Testing, Charles River); the endotoxin level was <5 EU per kg dosing. The total number of reformatted antibodies for each library is listed in Table 1. The positive control antibody used in this study was the biosimilar sirexatamab, a humanized monoclonal antibody targeting hDKK1 that is in phase 2 clinical development (Abeomics, #12-9195).

### Surface plasmon resonance (SPR) kinetics

High-throughput kinetic analysis was performed via a direct coupling kinetic format using a Carterra LSA SPR biosensor. An HC30M chip was first activated by EDC/S-NHS diluted in 0.1 M MES (pH 5.5) for 10 min. The antibodies were diluted to 4 µg/mL in 10 mM NaOAc (pH 4.5) and amine-coupled to the chip for 15 min, followed by quenching for 7 min in 1 M ethanolamine (pH 8.5) to deactivate the surface. Analytes were injected across an increasing 7-point concentration gradient at 25 °C in HEPES buffered saline Tween-20 EDTA (HBSTE, pH 7.4) with 1 mg/mL BSA, followed by a 5 min association and a 10 min dissociation period. The following reagents were commercially sourced: hDKK1 (Acro Biosystems, #DK1-H5221 and R&D Systems, #5439-DK), hDKK2 (R&D Systems, #6628-DK*)*, hDKK3 (R&D Systems #1118-DK), DKK4 (R&D Systems, #1269-DK), cyDKK1 (R&D Systems, #10450-DK), and mDKK1 (Acro Biosystems, #DK1-M52H6). DKK1-CRD1-Fc and DKK1-CRD2-Fc are produced internally. The data were analyzed using a 1:1 binding model in Carterra Kinetics software.

### SPR epitope binning

Epitope binning was conducted using the classical binning format. An HC30M chip was first activated by EDC/S-NHS diluted in 0.1 M MES (pH 5.5) for 10 min. The antibodies were diluted to 4 µg/mL in 10 mM NaOAc (pH 4.5) and amine-coupled to the chip for 15 min, followed by a 1 M ethanolamine (pH 8.5) quench for 7 min to deactivate the surface. Prior kinetic experiments demonstrated that the antibodies reliably bound to hDKK1 at a concentration of 60 nM. Thus, an injection of 60 nM of analyte hDKK1 (Acro Biosystems, #DK1-H5221) in HBSTE (pH 7.4) with 1 mg/mL BSA was introduced into the immobilized ligand antibody for 8 min, followed by a 250 nM injection of analyte antibody in HBSTE (pH 7.4) with 1 mg/mL BSA for 5 min. The surface was regenerated for two cycles of 30 seconds with IgG Elution Buffer (ThermoFisher Scientific #21004). Data were analyzed using Carterra Epitope software. The competition thresholds were adjusted based on the relative binding responses to the hDKK1 analyte alone. A heat map was constructed to represent the various interaction types between the analyte/ligand pairs. Green represents non-competitive interaction and red represents competitive interaction. Antibodies that exhibited similar patterns of interaction were grouped together to form bins.

### Epitope mapping

#### Sample preparation, PLIMB treatment and LC-MS/MS analysis

DKK1 solutions with experimental or control antibodies were prepared at a concentration of 1 mg/mL. Samples were prepared in 50 µL volumes and incubated at room temperature for 45 min to promote binding. For standard PLIMB labeling, four replicates of each condition were exposed to the PLIMB treatment for 20 s. For trifluoromethylation labeling, samples were treated with PLIMB in the presence of 50 mM sodium triflinate and 10 mM hydrogen peroxide. Following PLIMB exposure, the samples were precipitated, chemically denatured, digested with trypsin platinum (1:10 trypsin to total protein in solution), and then with elastase, and prepared for mass spectrometric analysis. The samples were analyzed using data-dependent acquisition with an Orbitrap Exploris 240 mass spectrometer.

#### Data analysis

The DKK1 sequences were searched using Protein Metrics Byos. The standard expected modifications and expected PLIMB modifications were used in the database search. Peptides were identified using MS and MS/MS spectra, setting a 1% false discovery rate (FDR) cut-off. The proportion of plasma-modified species was calculated based on the extracted ion chromatogram (XIC) relative peak areas of modified versus total peptide signals, including modified and unmodified peptides. The percent fold-change in modification was calculated for the DKK1 peptides in the bound and unbound states. The positive control antibody was directly compared with a control sample containing NIST-mAb. NIST-mAb is a standard monoclonal antibody produced by the National Institute of Standards and Technology (NIST) and is used as a non-binding isotype control. The Student’s t-test was performed to determine statistical significance [22].

### Wnt canonical signaling reporter assay

Wnt signaling activation was detected using the TCF/LEF reporter HEK293 cell line (BPS Bioscience, #60501). The cell line contains a firefly luciferase gene under the control of TCF/LEF responsive elements stably integrated into HEK293 cells. It is validated for the response to the stimulation of mouse and human WNT3a and to the treatment with an inhibitor of Wnt/ß-catenin signaling pathway. Cells were grown in Growth Medium 1B (BPS Bioscience, #79531) and seeded at 3.5 ×104 cells/well in 96-well cell culture plates (Corning #3610) overnight. On Day 2, mWNT3a (R&D Systems, #1324-WN, 50 ng/mL) and hDKK1 (R&D Systems, #5439-DK/CF, 500 ng/mL) were incubated with anti-DKK1 antibodies in assay buffer (BPS Bioscience Thaw medium 1, #60187 with 10 mM LiCl) at 37 °C for 30 min. The mixture was added to the cells and incubated for 30 min, followed by incubation with mWNT3a (50 ng/mL) for 5 h. The ONE-Step Luciferase Assay System (BPS Bioscience, #60690) was used to detect the TCF/LEF reporter signal.

### Osteoblast differentiation and mineralization assay

MC3T3-E1 mouse pre-osteoblast cells (ATCC, #CRL-2593) were cultured in minimum essential medium (MEM; Gibco, #A1049001) supplemented with 10% fetal bovine serum. For osteogenic differentiation, cells were seeded in 24-well plates at 5 × 10^4^ cells per well in MEM medium (Gibco, #12571063) containing ascorbic acid supplemented with 10% fetal bovine serum and 10 mM β-glycerol phosphate. Cultures were treated continuously with 1 µg/mL purified recombinant human DKK1 (BioLegend, #778602) and 100 nM anti-DKK1 antibodies for 14 days. The level of mineralization was assessed by measuring the extracellular calcium levels using Alizarin Red staining (Millipore, #ECM810).

### Wnt non-canonical JNK phosphorylation assay

The ability of the antibody hits to restore JNK phosphorylation was assayed using a JNK 1/2 ELISA Kit (Abcam, # ab176662). In brief, human Colo205 colon cancer cells were seeded at 1.0 ×105 cells per well under serum-free starvation conditions and incubated overnight at 37 °C. After removing the media the next day, hDKK1 (1 µg/mL) was incubated with anti-DKK1 antibodies ranging from to 0–100 nM for 30 min, and the mixtures were then added to the cells. Human WNT5a (200 ng/mL) was added to the cells for 30 min at 37 °C to induce Wnt non-canonical signaling. After incubation for 2 h at 37 °C, intracellular JNK phosphorylation status was determined using cell extracts for ELISA.

### GM-CSF release assay

The ability of the antibody hits to restore GM-CSF release from activated NK cells in human PBMCs (Stemcell, #70025) or human NK cells (Stemcell, #70036) was assayed using the ELISA MAX™ Deluxe Set Human GM-CSF Kit (BioLegend, #432004). Briefly, hDKK1 (500 ng/mL) was incubated with anti-DKK1 antibodies in RPMI supplemented with 10% FBS at 37 °C for 30 min. The mixtures were then added to human PBMCs seeded at 2.0 ×105 cells per well in 96-well plates (Corning), and incubated for 30 min at 37 °C. Mouse WNT3a (50 ng/mL) was added to the cells and incubated for 30 min at 37 °C. Human T-Activator CD3/CD28 Dynabeads (Invitrogen #11161D) were then added to 1 μl of beads per 4 × 10^6^ cells, or IL2 (100 U/mL) and IL15 (10 ng/mL) were added to activate the primary immune cell response. After incubation for 3 days at 37 °C, the culture media were harvested and used directly for GM-CSF ELISA according to the manufacturer’s instructions.

### Tumor cytotoxicity assay

The ability of antibody hits to promote tumor killing by human PBMCs was monitored by measuring the remaining tumor cell viability after co-culturing with immune cells. Briefly, hDKK1 (500 ng/mL) was incubated with anti-DKK1 antibodies in RPMI (pH 7.4) supplemented with 10% FBS at 37 °C for 30 min. The mixtures were then added to the co-culture of human PBMCs seeded at 4.0 × 105 cells with human PC3 prostatic cancer cells (ATCC, # CRL-1435) seeded at 5.0 × 103 cells per well in 96-well plates and incubated for 30 min at 37 °C. Mouse WNT3a (100 ng/mL) was added to the cells and incubated for 30 min at 37 °C. Human T-Activator CD3/CD28 Dynabeads were then added to 1 μl of beads per 4 × 10^6^ cells, or IL2 (100 U/mL) and IL15 (10 ng/mL) were added to activate the primary immune cell response. After incubation for 6 days at 37 °C, PBMCs and culture media were removed. The viability of the remaining adherent PC3 cells was determined using the CellTiter-Glo Cell Viability Assay (Promega, # G9681).

In addition to PC3 prostate cancer cell, cancer cell lines related to breast cancer (SKBR3), gastric adenocarcinoma (NCI-N87) and colon cancer (Colo-205) were selected for evaluating the lead anti-DKK1 antibodies in this cytotoxicity assay to strengthen the clinical translational value. The assay was performed with DiscoverX KILR assay (DiscoverX, # 497-1002P018, # 497-1004P021, # 497-1034P045). The KILR platform is based on the Enzyme-Fragment Complementation (EFC) technology, which uses two recombinant β-galactosidase (β-gal) enzyme fragments that act as an enzyme acceptor (EA) and an enzyme donor (ED). Separately, the fragments are inactive, but when combined, they form an active β-gal enzyme that hydrolyzes its substrate to produce a chemiluminescence signal. The cytotoxicity level is evaluated by co-culture of effector cells with target cells and treatment with candidate antibodies. Briefly, hDKK1 (500 ng/mL) was incubated with anti-DKK1 antibodies at 37 °C for 30 min. The mixtures were then added to the co-culture of activated effector T cells seeded at 5.0 × 104 cells per well with KILR cancer cells seeded at 5.0 × 103 cells per well in 96-well plates and incubated for 30 min at 37 °C. Mouse WNT3a (100 ng/mL) was added to the cells and incubated for 30 min at 37 °C. After incubation for 1 day at 37 °C, the KILR detection reagent was added, and the measurement of luminescence represented the cytotoxicity level.

### In vivo SCID-PC3 mouse model

Anti-DKK1 antibodies were tested for in vivo tumor-suppressing activity in HuCD34-NCG mice (Jackson Laboratory) inoculated with PC3 cells. Eight-week-old female SCID (CBySmn.Cg-Prkdcscid/J) mice were subcutaneously injected with 1 × 10^6^ PC3 cells in 100 μL serum-free medium per mouse (*n* = 6). Thirty-six mice were randomized into six groups when the mean tumor volume (TV) reached approximately 100 mm^3^ (36 days post-inoculation). The day on which randomization and treatment were initiated was represented as day 0. Treatments were administered by IP injection with eight doses (Q3D × 8 on days 0, 3, 6, 9, 12, 15, 18, and 21) for all groups. Survival bleeds via submandibular collection were obtained on days 7 (24 h after 3rd dose) and 22 (24 h after the last dose) from the animals in the study. When the mean TV of the isotype control group reached approximately 1000 mm^3^ on day 36, the animals were euthanized. Whole blood collected in an EDTA-coated tube was placed on ice for immune-profiling, and tumors were collected, weighed, and discarded. All data was analyzed using GraphPad software Prism 9 where *p* < 0.05 is considered statistically significant.

### In vivo toxicity evaluation in B-hDKK1 mice

B-hDKK1 mice (Biocytogen) were used to evaluate the potential toxicity of the test samples upon administration (*n* = 3). Body weight was measured, and clinical observations were performed twice weekly over the course of the study. Terminal plasma and serum samples were collected and shipped to the IDEXX for liver chemistry analysis. The overall health of animals was evaluated based on body weight (BW), a short toxicity panel analysis on day 18, a terminal expanded liver toxicity panel analysis on day 24 of the study, and the complete blood count tested periodically over the course of the study.

## Results

### Triage strategy for anti-DKK1 lead identification

We triaged anti-DKK1 candidate antibodies using a series of in vitro binding and functional assays and an in vivo efficacy study. VHH-Fc antibodies produced from our VHH synthetic libraries and machine learning platform were analyzed by SPR, epitope binning, and epitope mapping to determine their binding profiles. We then determined the function of the top performing binders using Wnt signaling reporter assays, immune cell activation, and tumor cell cytotoxicity assays. Tumor suppressive efficacy was determined using a xenograft in vivo study. Qualitatively, the libraries panned against biotinylated hDKK1 yielded hits in each assay as summarized in Table 1.

### SPR epitope binning and kinetics

**Fig. 2:**
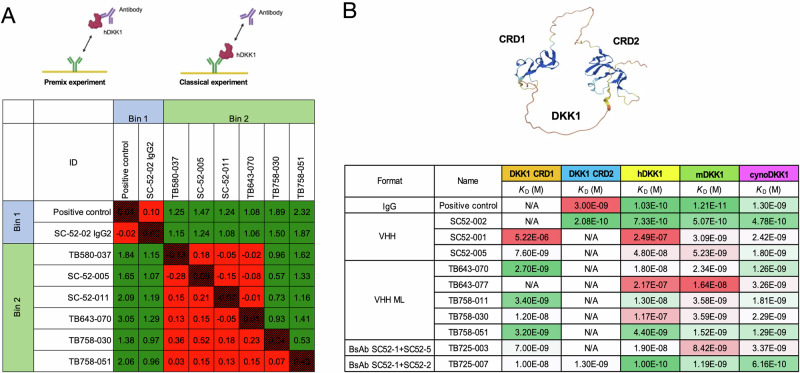
Anti-DKK1 antibodies binding to DKK1 by SPR analysis. **A** Two epitope bins are apparent amongst the Twist anti-DKK1 leads from this epitope binning analysis. The formation of Antibody-Antigen-Antibody complexes indicates the antibodies are not binding to the same epitope of DKK1. **B** Anti-DKK1 lead antibodies bind to hDKK1 cysteine-rich domain CRD1 or CRD2 or both CRD1 and CDR2 (in the instance of bispecific antibodies), and cross-reactivity with mouse and cynomolgus monkey DKK1. The assays were repeated in triplicate.

Antibody candidates were evaluated using high-throughput SPR to measure their kinetic affinity. The candidates exhibited a variety of affinities to hDKK1, ranging from nanomolar to picomolar affinities. A subset of antibodies was selected from the kinetics assay and matrixed in a cross-competition classical epitope binning assay using SPR. DKK1 binders were split into two bins (Fig. 2A). SC52-002 and DKK1 clinical stage control antibody were grouped together in the same competition bin (bin 1). SC52-005, SC52-011, TB643-070, TB758-030, and TB758-051 binned together in a second competition bin (bin 2). We further investigated the diversity of epitopes suggested by the epitope binning assay by exploring the binding kinetics of the antibodies to the cysteine-rich N-terminal (CRD1) and C-terminal (CRD2) regions of hDKK1. We found that the antibodies that belonged to bin 1 also bound to CRD2-Fc, whereas the antibodies that belonged to bin 2 bound to CRD1-Fc. Several candidates also showed cross reactivity with cynomolgus monkey DKK1 and mouse DKK1 (Fig. 2B). The assay was replicated three times.

### Epitope mapping showing anti-DKK1 binding to DKK1 CRD1 or CRD2

**Fig. 3:**
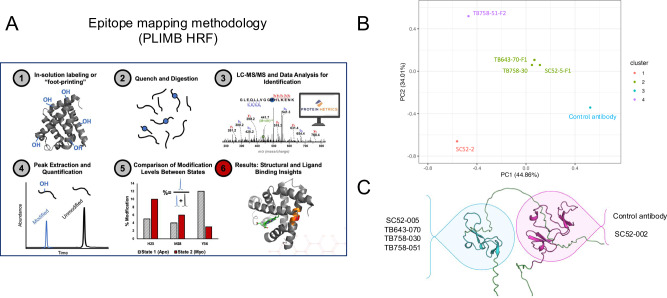
Epitope mapping of anti-DKK1 lead antibodies to hDKK1. **A** Epitope Mapping methodology: Differences in solvent accessibility of various regions of the antigen (DKK1) using PLIMB and hydroxyl radical labeling were measured. Each antibody was added individually at a 1:1 antibody/antigen molar ratio. For epitope mapping, peptide level-analysis shows the detection of larger-order structural changes and identification of regions of interest showing “protection” and “deprotection” upon complexation. Peptides with a decreased level of modification upon binding with the antibody indicate “protection”. Peptides with an increased level of modification upon complexation represent “deprotection” or areas of conformational change, which take place due to antibody binding. The peptides of interest were further validated by visually inspecting MS2 spectra and adjusting extracted ion chromatogram (XIC) windows across peaks that showed consistency between samples and whose modifications could be identified with MS2 hits. The peptide-level analysis was utilized for peptides in the candidate epitope regions to determine which of the individually labeled amino acids show the greatest changes in solvent accessibility due to binding. **B** Principal component analysis (PCA) of all quantified log2 fold-changes between unbound and bound states of all eight antibodies. Colors are representative of clusters as determined by partition around medoids (PAM). **C** Structural representation of DKK1 epitope groups. Blue: N-terminal cysteine-rich domain, Pink: C-terminal cysteine-rich domain, Green: unstructured. Structure from AlphaFold (AF-O94907-F1).

Potential peptide level epitopes were identified for eight candidate antibodies, utilizing the plasma-induced modification of biomolecule hydroxyl radical footprinting (PLIMB-HRF) method of epitope mapping. Differences in solvent accessibility of various regions of the antigen (DKK1) using PLIMB and hydroxyl radical labeling were measured. Each antibody was added individually at a 1:1 antibody/antigen molar ratio. For epitope mapping, peptide level-analysis shows the detection of larger-order structural changes and identification of regions of interest showing “protection” and “deprotection” upon complexation. Peptides with a decreased level of modification upon binding with the antibody indicate “protection”. Peptides with an increased level of modification upon complexation represent “deprotection” or areas of conformational change, which take place due to antibody binding. The peptides of interest were further validated by visually inspecting MS2 spectra and adjusting extracted ion chromatogram (XIC) windows across peaks that showed consistency between samples and whose modifications could be identified with MS2 hits. The peptide-level analysis was utilized for peptides in the candidate epitope regions to determine which of the individually labeled amino acids show the greatest changes in solvent accessibility due to binding (Fig. 3A). SC52-005, TB643-070, TB758-030, and TB758-051 bound to the N-terminal cysteine-rich domain (CRD1), while the clinical stage control antibody bound to a distinctly different region, the C-terminal cysteine-rich domain (CRD2) (Fig. 3B, C). CRD1-binding antibodies exhibited a potential hotspot for the epitope region of [G103-R115]. The CRD2-binding clinical stage control antibody exhibited a potential epitope at [H204-K208] and [G227-R236], whereas the CRD2-binding antibody SC52-002 exhibited candidate epitopes at [H204-K208] and [C237-R246]. SC52-002 also exhibited deprotection at [G227-R236], unlike the protection afforded by the control antibody at this position. This was further evidence that SC52-002 binds to a different epitope on DKK1 CDR2 compared to the positive control antibody (Supplementary 1). The unique epitopes identified by PLIMB-HRF mapping techniques provide potential explanations for the diversity in binding characteristics and functions identified from kinetics characterization, binning analysis, and cell-based assays.

### hDKK1 CRD2 binders block DKK1-receptor interaction and restore Wnt canonical signaling

**Fig. 4:**
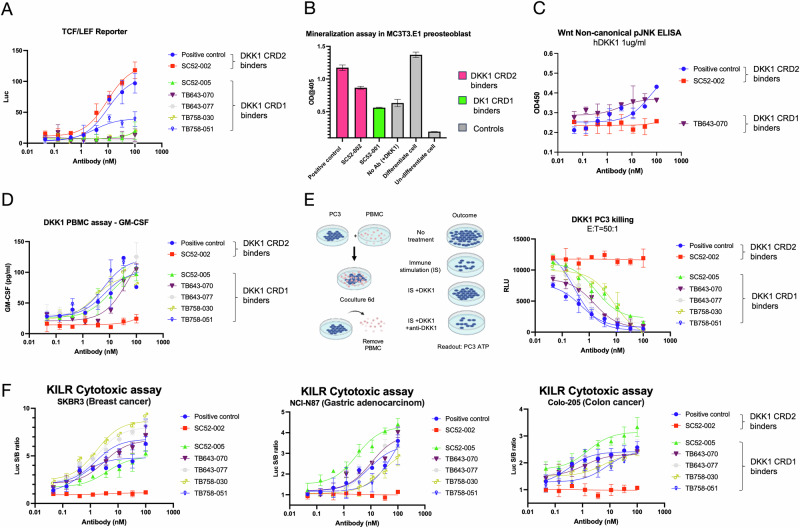
In vitro functional cell-based assay validation. **A** Wnt TCF/LEF reporter assay screening. Wnt TCF/LEF signaling is blocked by DKK1 binding to LRP5/6. Anti-DKK1 antibodies that bind to hDKK1 CRD2 block the binding of DKK1 to the co-receptors, and lead to the reactivation of Wnt canonical signaling. **B** MC3T3.E1 cell differentiation detection by mineralization assay. Soluble hDKK1 suppresses pre-osteoblast cell differentiation via the Wnt canonical pathway. Anti-DKK1 antibodies that bind to hDKK1 CRD2 block the binding of DKK1 to the LRP5/6 co-receptors and restore cell differentiation. **C** Wnt non-canonical phospho-JNK detection. Colo205 cells were treated with Wnt, DKK1, and anti-DKK1 lead antibodies. Intracellular JNK phosphorylation level was detected with ELISA. **D** Primary immune cell activation. DKK1 leads to immune suppression including T cell inactivation, MDSC accumulation, and NK cell clearance. GM-CSF is the biomarker for NK cell activation. Human PBMC were treated with an immune stimulator, mWNT3a, hDKK1, and DKK1 lead antibodies. Cytokine release of GM-CSF was measured by ELISA. Antibodies binding to CRD1 of DKK1 showed stronger NK cell activation. **E** PC3 tumor cell cytotoxicity by activated immune cells. T cells and NK cells in human PBMC were activated and co-cultured with PC3 tumor cells for 6 days. Activated immune cells kill PC3 cells, while hDKK1 treatment inhibits cytotoxicity. Blocking the interaction of hDKK1 to the receptor with Twist DKK1 lead antibodies restores the cytotoxicity potency. Antibodies binding to CRD1 of DKK1 showed stronger cytotoxicity. **F** Anti-DKK1 antibody targeting DKK1 CRD1 also induced cytotoxicity in breast, gastric and colon cancer cells. Using the KILR cytotoxicity assay, a high luminescence signal was detected in cytotoxic cells. All the samples were duplicated, and the assays were repeated three times.

The Wnt canonical pathway is activated by Wnt binding to the LRP5/6 co-receptor, which leads to the translocation of β-catenin into the nucleus. T-cell factor/lymphoid enhancer factor (TCF/LEF) transcription factor is activated by β-catenin. DKK1 inhibits β-catenin-dependent Wnt signaling, resulting in β-catenin degradation. The TEF/LEF luciferase reporter cell assay system served as a tool for detecting Wnt activation. In the presence of Wnt and DKK1, candidate antibodies SC52-002 and the positive control antibody (both of which bind to hDKK1 CRD2) blocked DKK1 binding to the LRP5/6 co-receptors, allowing Wnt binding to restore TCF/LEF signaling. Binding of candidate antibodies to hDKK1 CRD1 had a negligible effect on Wnt signaling restoration (Fig. 4A). Wnt ligands also stimulate bone growth, suggesting a strong regulatory role for the canonical Wnt signaling pathway in bone healing and highlighting its potential as a therapeutic strategy to augment fracture healing [23]. Osteoclasts erode the bone matrix by secreting acids and hydrolases to dissolve bone minerals and to digest organic components. Osteoblasts arise from undifferentiated precursor cells and deposit a mineralized matrix consisting of collagen, calcium, and phosphorus, leading to the formation of new bone. The balance between the activity of osteoblasts and osteoclasts determines the mass and density of bones [24]. To study osteoblast differentiation, mouse pre-osteoblast MC3T3-E1 cells were used for high osteoblast differentiation and mineralization after growth in a medium containing ascorbic acid. The differentiated cells were treated continuously with human DKK1 and antibodies for 14 days, and extracellular calcium was measured by Alizarin Red staining. Calcium forms an Alizarin Red S-calcium complex during the chelation process, and the end product is a bright red stain. Alizarin red is a commonly used stain to identify calcium-containing osteocytes in differentiated cultures [25]. The results showed that SC52-002 and the positive control antibody, which binds to DKK1 CRD2, could restore osteoblast differentiation inhibited by DKK1. Candidate antibodies binding to hDKK1 CRD1 had a negligible effect on the restoration of cell differentiation (Fig. 4B). The data indicate that hDKK1 CRD2 is involved in the Wnt canonical pathway, which agrees with previous research [26]. All the samples were loaded with duplication, and the assays were repeated three times.

### hDKK1 CRD1 binders activate NK cells and promote cytotoxicity on tumor cells via Wnt non-canonical pathway

One of the Wnt non-canonical signaling pathways involves RhoA, Rac GTPases, and c-Jun N-terminal kinase (JNK). Detection of JNK phosphorylation in the presence of Wnt and DKK1 indicated the restoration of the Wnt non-canonical pathway by anti-DKK1 antibodies. The data demonstrated that the antibodies binding to DKK1 CRD1 restored JNK phosphorylation (Fig. 4C). Several reports have shown that NK cells play important roles in suppressing tumor growth during DKK1-mediated immunosuppression and cancer progression [11, 27]. In this study, we developed an in vitro primary immune cell assay by detecting NK cell activation to evaluate the effect of candidate antibodies on immune response. In the presence of Wnt, DKK1, and immune cell stimulators, hDKK1 CRD1 binders SC52-005, TB643-070, TB758-030, and TB758-051 induced NK cell activation, as evaluated by GM-CSF secretion. Meanwhile, the hDKK1 CRD2 binder SC52-002 showed no effect on NK cell activation. Antibodies binding to the CRD1 of DKK1 showed stronger NK cell activation (Fig. 4D). Primary immune cells were co-cultured with PC3 human prostate cancer cells in the presence of Wnt, DKK1, or immune stimulators. The results indicated that the hDKK1 CRD1 binders SC52-005, TB643-070, TB758-030, and TB758-051 demonstrated cytotoxicity in PC3 cells, whereas the hDKK1 CRD2 binder, SC52-002, had no effect (Fig. 4E). In addition to PC3 prostate cancer cell, cancer cell lines related to breast cancer (SKBR3), gastric adenocarcinoma (NCI-N87) and colon cancer (Colo-205) were selected for evaluation of the DKK1 antibodies in the cytotoxicity assay to strengthen the clinical translational value. The assay was performed with DiscoverX KILR assay. The results consistently demonstrated that antibodies targeting hDKK1 CRD1 led to an activated T cell response and consequently cell cytotoxicity in breast cancer (SKBR3), gastric adenocarcinoma (NCI-N87) and colon cancer (Colo-205) cells, whereas the hDKK1 CRD2 binder, SC52-002, had no effect (Fig. 4F). The antibodies that target DKK1 CRD1 are involved in the immune response activation and inhibit tumor growth in all four tumor lines, indicating its potential universal role in cancer therapy. These results suggest that DKK1 CRD2 is involved in the Wnt canonical pathway, whereas DKK1 CRD1 is involved in the Wnt non-canonical pathway, leading to immune cell activation and tumor cell cytotoxicity. The positive control antibody is an hDKK1 CRD2 binder, but it possesses the ability to activate immune cells, unlike SC52-002. This may reflect their binding to different epitopes on hDKK1 CRD2 (Fig. 3B). These results indicated that antibodies binding to different epitopes of hDKK1 lead to different biological functions. All the samples were loaded with duplication, and the assays were repeated three times.

### Bispecific antibody binding to both DKK1 CRD1 and 2 activates both Wnt signaling and the immune response

**Fig. 5:**
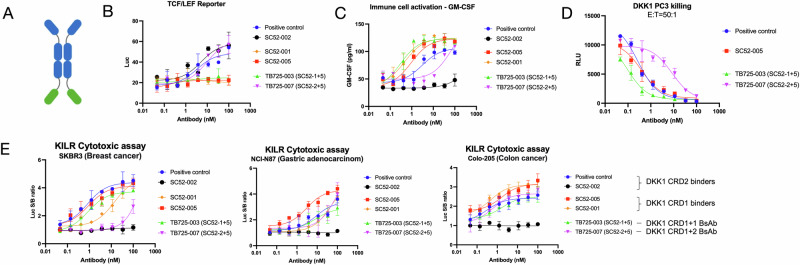
Evaluation of bispecific antibody format in cell-based functional assay. **A** SC52-001 and SC52-005 bind to DKK1 CRD1 and elicit immune cell activation, while SC52-2 binds to DKK1 CRD2 and activates Wnt signaling. Bispecific antibodies consisting of a combination of SC52-001, 002, or 005 were generated as shown. **B** The potencies of Wnt signal activation of DKK1 CRD1 binders are increased with bispecific antibody treatment that combine DKK1 CRD2 binders as compared to that of monospecific antibody treatment. **C** NK cell activation of DKK1 CRD2 binders increased with bispecific antibody treatment that combine DKK1 CRD1 binders as compared to that of monospecific antibody treatment. **D**, **E** Tumor cell cytotoxicity effect is upregulated in DKK1 CRD2 binders with bispecific antibody treatment that combine DKK1 CRD1 binders as compared to that of monospecific antibody treatment. All the samples were duplicated, and the assays were repeated twice.

SC52-001 and SC52-005 bind to DKK1 CRD1 and activate the immune response, whereas SC52-002 binds to DKK1 CRD2 and activates Wnt signaling. Bispecific antibodies comprising a combination of SC52-001, 002, or 005 were generated, as depicted in Fig. 5A. TB725-003 consists of a combination of SC52-001 and SC52-005, which bind only to hDKK1 CRD1, whereas TB725-007 consists of a combination of SC52-002 and SC52-005, which bind to both the CRD1 and CRD2 of hDKK1. With the ability to bind both hDKK1 CRD1 and CRD2, TB725-007 was capable of both Wnt signal restoration (Fig. 5B) and immune cell activation (Fig. 5C). These data further confirmed that hDKK1 CRD1 plays an important role in the immune response, and CRD2 is involved in Wnt canonical signaling. In the PC3 tumor cytotoxicity assay, TB725-003 demonstrated robust tumor killing activity, indicating that the addition of binding to hDKK1 CRD1 in a quadrivalent bispecific antibody format can improve tumor suppression (Fig. 5D). In the KILR tumor cytotoxicity assays applied to SKBR3, NCI-N87, and Colo-205 tumor cells, bispecific antibody TB725-007 combining DKK1 CRD1 and CRD2 binders increased the cytotoxicity effect compared to that of DKK1 CRD2 binder SC52-002 alone (Fig. 5E). The results suggest that DKK1 CRD1 binders are involved in immune responses, while DKK1 CRD2 binders regulate Wnt canonical signaling. The combination of DKK1 CRD1 and CRD2 binding led to activation of both pathways. All the samples were loaded in duplication and the assays were repeated twice.

### Anti-DKK1 antibodies binding to hDKK1 CRD1 suppress tumor growth in xenograft model

**Fig. 6:**
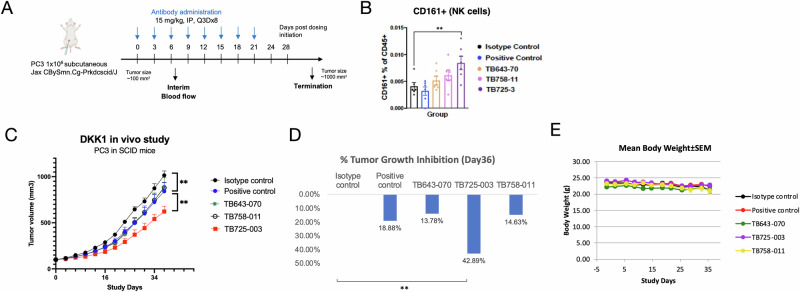
Anti-DKK1 lead antibodies cause inhibition of tumor growth. **A** The homozygous SCID mice were inoculated with PC3 cells. Dosing was initiated at a tumor volume average of ~100 mm^3^ with 10 mg/kg antibody via intraperitoneal injection once every 3 days for 8 cycles (Q3D x 8). Tumor sizes were measured 3 times a week (*n* = 6). **B** The frequency of CD161^+^ cells within the CD45^+^ cell population was measured on Day 7 post-administration. The number of NK cells increases upon TB725-003 treatment. **C** Tumor size measurement indicates TB725-003 treatment significantly suppresses tumor growth, demonstrating their efficacy in tumor suppression. No significant difference is observed with mice body weight changes. **D** Tumor growth inhibition shows 42.89% reduction in size upon TB725-003 treatment, respectively, while the positive control antibody gave 18.88% inhibition. Tumor Growth Inhibition (TGI, %) = [1−(T t − T 0)/(C t − C 0)] × 100%, where T t = mean TV of treated at time t, T 0 = mean TV of treated at time 0 (baseline), Ct = mean TV of control at time t and C 0 = mean TV of control at time 0 (baseline). **E** No significant changes are observed for mice body weight measurements. **p ≤ 0.01, ***p ≤ 0.001 vs. isotype control by Kruskal–Walli’s test. The study was repeated in triplicate.

Previous studies have shown that DKK1 blockade can slow tumor growth in an NK cell-dependent manner [11], and our data also support the notion that DKK1 blockade leads to NK cell activation. To assess the contribution of NK cells to DKK1-mediated tumor growth in vivo, we used the DKK1-expressing human prostate cancer cell line PC3 in a severe combined immunodeficient (SCID) mouse model. SCID mice have genetic immune deficiency that affects B and T cells. Due to the lack of mature B and T lymphocytes but normal NK cell function, these mouse models are ideal for xeno-engraftment of human cells and tissues. Having established that DKK1 CRD1 binders suppressed tumor growth in vitro, we tested their efficacy in this xenograft model (Fig. 6A). The frequency of CD161^+^ cells within the CD45^+^ cell population was measured on day 7 post-administration, and the number of NK cells increased after TB725-003 treatment (Fig. 6B). Treatment with TB725-003 led to a statistically significant reduction in tumor growth compared with the positive control (*p* < 0.01) and isotype control (*p* < 0.001) after 36 days of antibody administration (Fig. 6C). TB725-003 yielded the most significant tumor reduction of 44.75% and 42.89%, respectively, while the positive control antibody elicited only 18.88% tumor reduction (Fig. 6D). The other treatment groups also demonstrated reductions in the tumor mass, but these were not statistically significant. No significant body weight loss (BWL) (>20%) was observed (Fig. 6E). Most mice experienced maximum BWL in the last 10 days of the study, which indicates that BWL might be due to tumor cachexia. No unexpected clinical observations or deaths occurred during the study. The study was performed in triplicate.

### Anti-DKK1 lead antibody TB643-070 does not generate in vivo toxicity in humanized DKK1 mice

**Fig. 7:**
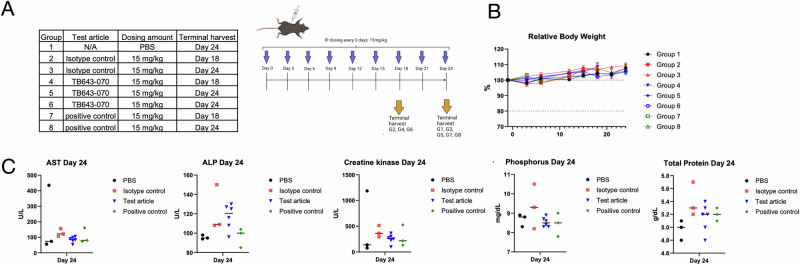
Toxicity evaluation in B-hDKK1 mice. **A** A schematic diagram of the study design (*n* = 3). **B** Relative mean body weight: The relative body weight at each measurement time point is plotted. A dotted line at 80% is plotted to identify the animals that experienced a body weight loss of 20% or more. Groups are color-coded by test sample: PBS (black), isotype control (red), TB643-070 (blue), and positive control (green). **C** Liver chemistry tests performed on Day 24 samples: Measurements for aspartate aminotransferase (AST), alkaline phosphatase (ALP), creatine kinase, phosphorus, and total protein were plotted from Day 24 terminal serum samples. All samples obtained from animals receiving the same treatment were combined into one group. TB643-070 shows no significant liver toxicity. Significant comparisons obtained from Tukey’s multiple comparisons test are displayed on each graph: no significance, *p* > 0.05.

Humanized DKK1 mice (B-hDKK1) were used to evaluate the potential toxicity caused by TB643-070 upon administration since the antibody induced 13.78% tumor suppression and was developed from our internal machine learning model. A short toxicity panel was analyzed on day 18 and a terminal expanded liver toxicity panel was analyzed on day 24 (Fig. 7A). Body weight remained stable after treatment compared to day 1. None of the mice experienced weight loss greater than 20% of the initial body weight, nor were any other adverse effects observed (Fig. 7B). None of the animals had to be sacrificed because they reached a humane endpoint. The liver chemistry data did not show any significant differences compared to PBS and the isotype control for the majority of the observed parameters, suggesting that TB643-070 did not cause in vivo toxicity in B- hDKK1 mice (Fig. 7C).

## Discussion

Wnt signaling is involved in developmental processes during embryogenesis, including cardiovascular development and neural development, as well as in regulating cell behaviors such as cell proliferation, migration, adhesion, and polarity. Abnormal regulation of this signaling cascade leads to different cancer and non-cancer diseases; consequently, the Wnt pathway has been the focus of drug discovery and immunotherapy. Dickkopf-1 (DKK1) is a well-characterized Wnt inhibitor and component of the Wnt/β-catenin signaling pathway, and its dysregulation is associated with multiple abnormal pathologies, including osteoporosis, Alzheimer’s disease, diabetes, and various cancers. Targeting of DKK1 in drug discovery has been an increasing area of interest in cancer therapy, with candidates progressing through clinical studies that are currently in phase 1 and phase 2 trials [28].

In this study, we discovered anti-DKK1 antibodies using synthetic libraries and machine learning methods and found that the antibodies binding to DKK1 CRD2 were capable of restoring the Wnt canonical pathway, whereas antibodies binding to DKK1 CRD1 could regulate Wnt non-canonical signaling, promote the immune cell response in vitro, and suppress prostate tumor growth in vivo. Recent DKK1 studies in prostate cancer have also demonstrated that tumor suppression can be independent of canonical Wnt signaling. [29, 30]. Targeting DKK1 has also been used as a potential treatment for different tumor types, including breast cancer [31, 32], non-small cell lung cancer (NSCLC) [33, 34], gastric cancer [35, 36], colorectal cancer [37, 38], and pancreatic cancer [39–41]. To further enhance efficacy for the inhibition of tumor growth, a combination treatment of an anti-DKK1 antibody with an anti-PD1 antibody has yielded promising clinical efficacy in clinical trials of cancer therapy [28, 42–44].

Our lead molecules have novel mechanisms of action and show in vivo single-agent efficacy, with ~25% more tumor suppression than sirexatamab. Sirexatamab binds to DKK1 CRD2 but behaves differently to another CRD2 binder, SC52-002, whereas the rest of the lead antibody panel binds to DKK1 CRD1. The differences in the mechanisms of action may be due to their different binding profiles, as shown in Fig. 3B. Recent DKK1 studies have reported that observed tumor suppression is independent of canonical Wnt signaling in prostate cancer [29, 30]. In prostate cancer cells, ovarian cancer cells, and osteosarcoma, JNK signaling is activated by DKK1 via a β-catenin-independent pathway and promotes cell invasion or tumor growth. The competitive binding of DKK1 to LRP5/6 could shift the Frizzled receptors to the JNK pathway because of the higher availability of Wnt and activation of JNK signaling [45]. LRP5 and LRP6 proteins are 70% identical and consist of an extracellular domain with four YWTD (Tyr–Trp–Thr–Asp)-type β-propeller motifs (P1–P4), four EGF-like domains (E1–E4), and three LDLR type A domains (L1–L3) close to the transmembrane region. While the P1E1P2E2 unit binds specifically WNT1, WNT2, WNT2b, WNT6 and WNT9b, P3E3P4E4 binds WNT3 and WNT3a [46, 47]. To antagonize LRP6 activity, DKK1 uses the CRD1 and CRD2 domains to interact with the P1 and P3 domains of LRP6, respectively, thereby directly competing for the binding of both classes of WNTs. The binding of WNT3a to the LRP6 P3 region is antagonized by the binding of DKK1 CRD2, which affects Wnt canonical signaling. Previous study reported that antibodies targeting the LRP5/6-P3 region inhibited WNT3/3a-mediated cellular responses. Furthermore, these anti-LRP5/6 VHHs block the growth of Wnt-hypersensitive Rnf43/Znrf3-mutant intestinal organoids [48]. Targeting LRP6 may provide a potential strategy for the treatment of Wnt-hypersensitive tumors; however, LRP6 can interact with multiple protein partners and act as a co-receptor for many other extracellular molecules, thereby potentially leading to the risk of side effects. In addition to the Wnt-dependent pathway, DKK1 can regulate cell proliferation via a Wnt-independent pathway. Cytoskeleton-associated protein 4 (CKAP4) was recently identified as another DKK1 receptor that binds to DKK1 CRD1. Binding of DKK1 to the CKAP4 receptor activates PI3K/Akt signaling and stimulates cell proliferation. When DKK1 binds to both LRP6 and CKAP4, cell proliferation is promoted compared with the binding of CKAP4 alone [49]. Pharmacological treatment with a rabbit anti-CKAP4 polyclonal antibody suppressed tumor formation in mice, indicating the potential of CKAP4 as another antitumor target linked to this pathway [50]. Another humanized monoclonal antibody, v1Lt1, has been shown to inhibit DKK1-CKAP4 signaling and AKT activity, as well as prevent sphere formation in pancreatic cancer cells and suppress xenograft tumor formation induced by human pancreatic cancer cells and tumor growth in murine cancer models utilizing orthotopic transplantation of murine pancreatic cancer organoids into the pancreas [51]. Furthermore, the DKK1/CKAP4 pathway upregulates the expression of plasmalemma vesicle-associated proteins in cholangiocarcinoma cells, which is positively linked to angiogenesis in various tumors [52]. DKK1 is overexpressed in multiple tumors and may be a potential biomarker. Our panel of VHH leads that bind to DKK1 CRD1 also show tumor suppression, whereas CKAP4 binding to DKK1 CRD1 leads to tumor progression. The mechanism of action of anti-DKK1 via Wnt-dependent or Wnt-independent pathways reinforces the potential of this target for anti-angiogenic cancer therapy.

Wnt signaling has been shown to promote bone formation, and inactivation of this pathway leads to osteopenic states [23]. Previous in vitro data showed that DKK1 inhibits cell differentiation and bone metabolism in MC3T3-E1 pre-osteoblastic cells, resulting in decreased bone mass and microstructure [53]. Preclinical and clinical research indicates that high DKK1 expression can impair osteoblast activity and cause bone loss because DKK1 blocks osteoblast differentiation and impedes bone formation [54, 55]. Fracture repair is promoted by DKK1 blockage, and a decrease in DKK1 gene expression leads to an increase in bone mass and strength [56]. Many bone diseases, including osteoporosis, a common phenomenon in postmenopausal women in which bone mass is greatly reduced, and osteogenesis imperfecta (OI), also known as brittle-bone disease, are likely caused by misregulation of osteoblasts and osteoclasts. Understanding the molecular mechanisms underlying osteogenesis and the process by which new bone is formed is of significant clinical importance [57]. Therefore, treatment with DKK1 antibody could be an effective means of increasing bone formation in bone loss diseases.

Sirexatamab (Leap Therapeutics), a humanized IgG4-κ antibody, has progressed to phase 2 clinical studies in multiple solid tumor types, including gastrointestinal tract-associated tumors and some reproductive cancer types, and several other anti-DKK1 antibody drugs have been utilized in both cancer and bone disease studies. BHQ880 (Novartis, Cambridge, MA, United States) is a phage-derived fully human DKK1 IgG1-λ antibody that neutralizes both human DKK1 and murine DKK1. Treatment with BHQ880 slowed the growth of orthotopically implanted patient-derived osteosarcoma xenografts and inhibited metastasis. This effect is correlated with increased expression of the bone differentiation marker osteopontin, showing that BHQ880 is an anti-metastatic agent for osteosarcoma in a preclinical model [58] and is in phase 2 clinical studies for the treatment of multiple myeloma. In addition, BHQ880 has recently been reported to improve motor functional recovery, promote preservation of myelinated tissue, and reduce astroglial and microglia/macrophage reactivity in a rat model of contusion spinal cord injury [59]. JS-015 (Shanghai Junshi Biosciences Co. Ltd.) is a humanized IgG4-κ antibody in phase 2 development for the treatment of esophageal squamous cell carcinoma, adenocarcinoma of the gastroesophageal junction, colorectal cancer, gastric cancer, and pancreatic ductal adenocarcinoma [ClinicalTrials.gov ID NCT06139211]. RH2-18 (Merck Research Laboratories) is a fully human IgG2-λ antibody isolated by screening a phage-displayed scFv library that recognizes only non-denatured native DKK1. RH2-18 induces systemic pharmacologic bone efficacy and new bone formation and also demonstrates a rate-limiting role of DKK1 at multiple skeletal sites in vivo [60] however, this antibody was terminated at a preclinical stage of development, similar to several other monospecific antibodies for DKK1, such as PF-04840082 [61]. Hetero-DS (Amgen), also known as AMG 147, is a bispecific antibody constructed on an IgG2 backbone [62], that targets sclerostin and DKK1 for the treatment of bone disorders, including fracture healing and bone formation. Hetero-DS/AMG 147 triggers a synergistic effect by the simultaneous blockade of DKK1 and sclerostin [63], but it no longer appears to be in development. AGA-2115 (Angitia Biopharmaceuticals) is also a humanized bispecific antibody that targets sclerostin and DKK1 which is in Phase 1 clinical development for the treatment of osteogenesis imperfecta. Given the positive outcome attained with setrusumab (an anti-sclerostin antibody) in adults with OI holding promise and progression to phase 3 studies in this condition [64], the value of a combination therapy holds potential for further clinical benefit in OI patients where sclerostin is another protein target associated with the Wnt signaling pathway. There have also been other DKK1 combinatorial therapies explored for their enhanced efficacy in treating oncology indications, such as DKK1/FcγR1 where the latter target acts as an internalizing effector protein [Regeneron, WO2017190079], or bone metabolism, such as DKK1/RANKL where the latter targets is also involved in the Wnt signaling pathway [Eli Lilly, WO2016186957]. Neutralizing anti-DKK1 antibodies also hold therapeutic potential in combination with chemotherapy as recently demonstrated with a functional reagent antibody, AF1096 (R&D Systems), where improved therapeutic efficacy of paclitaxel in two murine models of breast cancer was reported in addition to the alleviation of paclitaxel-induced peripheral neuropathy [59].

In summary, the available preclinical and clinical data to date support the inhibition of DKK1 by our lead antibody panel as a therapeutic strategy for the treatment of cancer and bone metabolic diseases. Our results demonstrated greater potency than the clinical stage control antibody without cross-interference between Wnt canonical and non-canonical pathways. The avoidance of cross-interference between these two pathways leads to a potentially lower risk of toxicity and side effects. This underscores the importance of epitope diversity, which can lead to different biological functions and the ability of machine learning to enable access to diversity. Moreover, current research suggests that other protein targets in the Wnt signaling pathway may hold potential as additional therapeutic strategies for combinatorial or bispecific interventions.

## Supplementary information

Supplementary 1

## Data Availability

All relevant data are within the paper and its Supporting Information files. Materials described in the manuscript will be freely available to any researcher wishing to use them for non-commercial purposes, without breaching participant confidentiality.
