# Supplementary material for: A multi-faceted discovery strategy identifies functional antibodies binding to cysteine-rich domain 1 of hDKK1 for cancer immunotherapy via Wnt non-canonical pathway
Source: Oncogene. 2025 May 20;44(31):2677–88. doi: 10.1038/s41388-025-03445-6 (PMC12301234; doi:10.1038/s41388-025-03445-6)
Supplement: Supplementary file 1 — Supplementary 1 [file 41388_2025_3445_MOESM1_ESM.docx]

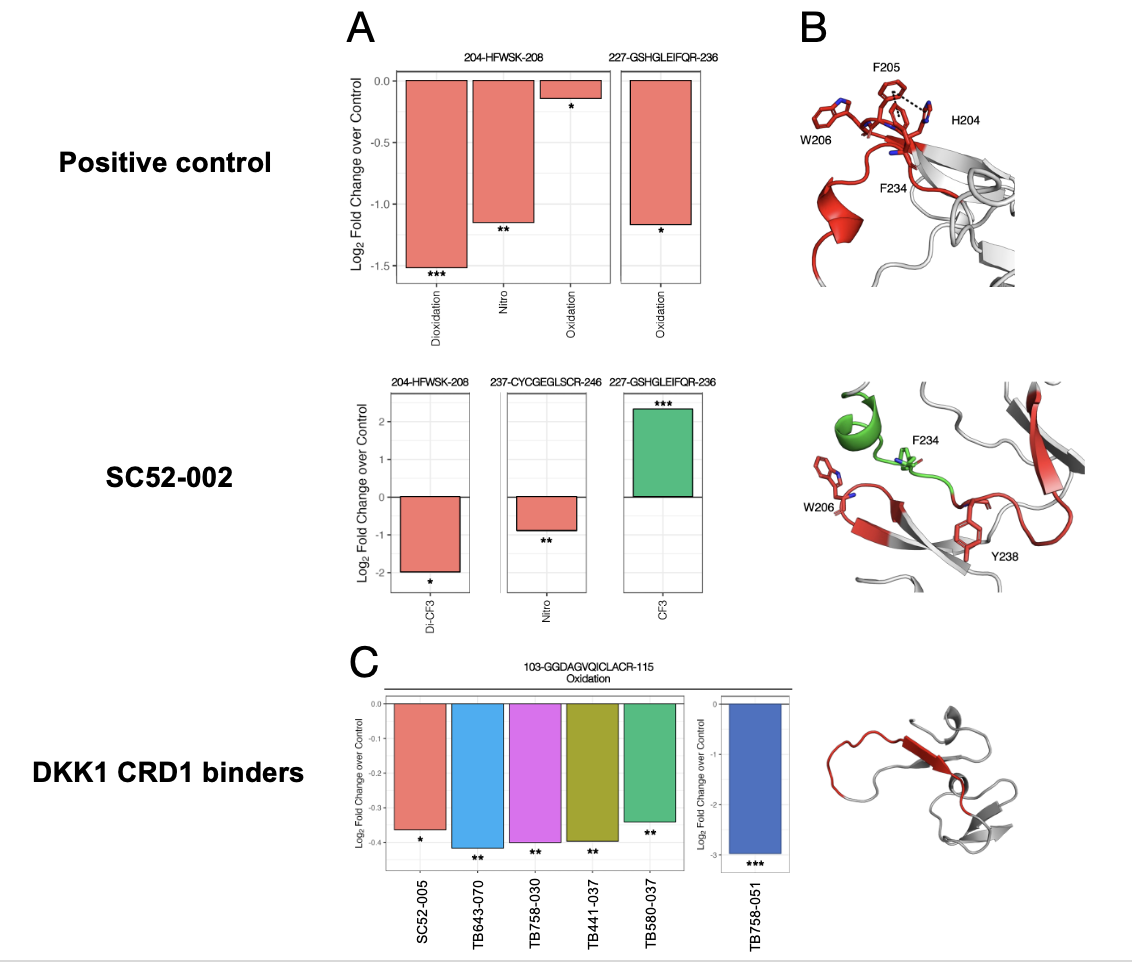


**Supplementary 1. Peptide-level DKK1 modification in candidate epitope region.** (A) Log2 fold-change was calculated from the percentage modification of DKK1 bound to the unbound control. Decreases in modification are shown in pink and increases in modification are shown in green. (B) The structure of DKK1-domain 2, CRD2, [C189-H264] is shown; peptides with significant protection are highlighted in red, and peptides with significant deprotection are shown in green. Amino acids that significantly contributed to peptide-level changes are shown as sticks. The Pi-pi interactions are indicated by dashed lines. (C) Log2 fold-change was calculated from the percentage modification of DKK1 bound to the unbound control. Colors represent individual antibodies. The structure of DKK1-domain 1, CRD1, [C86-S140] is shown, and peptides with significant protection are highlighted in red. Statistical significance was calculated via t-tests between the two conditions, bound or unbound, by asterisk (*p<0.05, **p<0.01, ***p<0.001, n=4 per condition). The residues were numbered using the human DKK1 UniProt canonical sequence. The structures were obtained from AlphaFold (AF-O94907-F1).
